# Determinants of waste generation in operating rooms

**DOI:** 10.1007/s00264-026-06763-w

**Published:** 2026-03-02

**Authors:** Annika Steinmeier, Nicole Feder, Robert Mahlow, Ulrich Stöckle, Oliver Birkelbach, Robert Zahn

**Affiliations:** 1 Centrum für Muskuloskeletale Chirurgie, Charité - Universitätsmedizin Berlin, Berlin, Germany; 2 Klinik für Orthopädie, Universitätsklinikum Ulm, Ulm, Germany

**Keywords:** Waste management, Sustainability, Clinical waste, Waste analysis, Waste reduction, Operating waste

## Abstract

**Purpose:**

Waste management in hospitals is important for environmental sustainability, as disposal of operations waste causes substantial greenhouse gas emissions. This study aimed to identify factors influencing waste generation in orthopaedics and traumatology.

**Methods:**

In this prospective study, the weight of waste and drapes from 272 orthopaedic and trauma operations was measured. Waste production was analyzed regarding to anatomical region, operation type, and duration.

**Results:**

Analysing all operations, the amount of waste differed significantly between anatomical regions (*p* < 0.001). When separating drapes, no significant differences between anatomical regions were found in waste, but in drapes (*p* < 0.001). The amount of waste differed significantly between operation types and correlated significantly with the operation duration (*p* < 0.001).

**Conclusion:**

Operating room waste is influenced by anatomical regions and the drapes required for it. Operation duration significantly increases the amount of waste. These findings can support the development of targeted strategies to reduce waste in operating rooms.

## Introduction

The healthcare system accounts for approximately 4.4% of greenhouse gas emissions and 8.5% of environmental impacts worldwide, thereby contributing substantially to climate change [1–4]. In addition, increasing air pollution an toxic changes to water and soil pose further threats to human health [5]. These developments result in extreme weather events, increased temperature and rising sea levels [4], all of which promote malnutrition, cardiovascular and pulmonary diseases, infections and vector-borne illnesses [2, 4, 6, 7]. Climate change thus represents a significant global health risk [8]. In response, the United Nations has adopted a strategy to reduce greenhouse gas emissions and environmental damage caused by the healthcare system. This plan emphasises the aspects reduction, recycling, reuse, use of renewable energies and research [5, 9].

Besides energy consumption and transportation logistics, waste management is particularly important in the healthcare system, with the German and American healthcare sectors each producing approximately up to six million tons of waste annually [1, 5, 10]. Within this context, orthopaedics and trauma surgery are responsible for more waste than any other medical specialty [11].

Approximately 30% of hospital waste is generated in operating rooms [3, 12]. Due to the potential of infectious contamination, this waste is often generally classified as hospital-specific waste that must be destroyed in specialised incinerators at temperatures above 1100 °C, which is associated with substantial greenhouse gas emissions [1, 13].

Optimizing operating room waste management is therefore a crucial component in the transition towards a more sustainable healthcare system. A precise analysis of waste generation within operating rooms is essential. Prior research has already shown that hip or knee arthroplasty procedures generate a particularly high amount of waste [13–15]. However, existing studies have so far focused on evaluating waste separation and recycling processes or analysed only small numbers of procedures. To enable a well-founded understanding of the waste generated, a differentiated assessment of influencing factors across various types of operations is required. The aim of this prospective study was therefore to compare waste quantities across different types of procedures and anatomical regions. The hypothesis was, that these factors significantly influence the amount of waste in operating rooms. Primary endpoints were the weight of waste and reusable drapes for different operations, anatomical regions and per time.

## Material and methods

In this prospective study, waste generated by 272 operations of a maximum-care and level I trauma centre at a university hospital was measured. All orthopaedic and trauma operations scheduled on regular working days were include in the survey, while unscheduled emergency operations were excluded. To determine the amount of waste, the weight of the waste was measured separately for each operation from the start of the preparations for the operation until the completion of the post-operative follow up tasks. The waste was categorised into potentially infectious hospital-specific waste and potentially recyclable materials according to established waste separation procedures. Paper and cardboard packaging disposed of outside the operation room and were not included in the survey. Reusable drapes, replacing disposable drapes, were used in 130 operations and their weight was measured separately. The duration, preparation and follow-up time of each operation were recorded in minutes.

Procedures were categorised into endoprosthetic operations, arthroscopic procedures, osteosynthesis, implant removal and others. Anatomical areas were classified into shoulder, upper extremity (distal to the shoulder), hip, lower extremity (distal to the hip), spine, and other areas. As this was an observational study, no ethical approval was required.

Descriptives statistics were calculated as mean values and standard deviations for interval-scaled variables. Depending on data distribution, t-tests, Mann-Whittney U tests, Kruskal–Wallis tests and post hoc tests according to Dunn-Bonferroni were applied. Nominal variables were analysed using absolute and relative frequencies and chi-square test was used for comparison. Statistical significance level was set at 0.05. IBM SPSS Statistics software (version 29.0.0) was used for statistical analysis.

## Results

A total of 272 operations were analysed over a period of 20 days. The average amount of waste per operation was 5.45 (± 2.31) kg. The average proportion of potentially recyclable material was 19.72%. The average operating time from incision to suture was 79.49 (± 49.86) minutes.

### Anatomical region of operation

Analysing all operations regarding the anatomical region, significant differences were observed in terms of total waste weight (x^2^(5) = 36.51; *p* < 0.001). Post hoc analysis identified significantly higher waste quantities in shoulder procedures compared to operations on the upper extremities distal to the shoulder (6.27 (± 2.20) kg vs. 4.42 (± 1.59) kg; *p* < 0.001) or lower extremity distal to the hip (6.27 (± 2.20) kg vs. 4.81 (± 1.82) kg; *p* = 0.001). Hip procedures also produced significantly more waste than operations on the upper extremity (6.67 (± 3.20) kg vs. 4.42 (± 1.59) kg; *p* < 0.001) or lower extremity distal to the hip (6.67 (± 3.20) vs. 4.81 (± 1.82) kg; p = 0.003). Spinal procedures generated more waste than upper extremity operations (6.17 (± 2.28) kg vs. 4.42 (± 1.59) kg; *p* = 0.029).

**Fig. 1:**
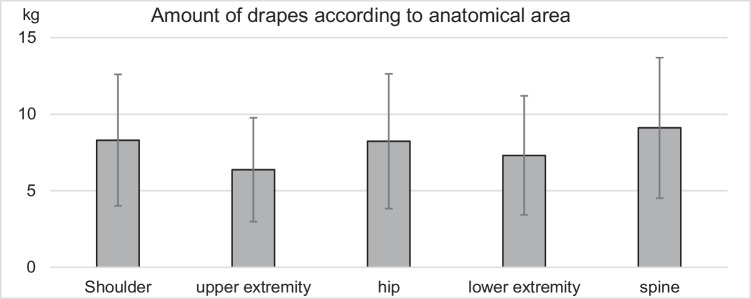
Amount of drapes according to anatomical area: The amount of reusable drapes showed significant differences between anatomical areas (x.^2^(5) = 26.39; *p* < 0.001)

When analysing only operations performed using reusable drapes, no significant differences in total waste (x^2^(5) = 10.61; *p* = 0.06) or hospital-specific waste were observed across anatomical regions (x^2^(5) = 8.49; *p* = 0.13) Fig. 1. However, the amount of reusable drapes differed significantly between anatomical region (x^2^(5) = 26.39; *p* < 0.001). Significantly fewer reusable drapes were measured for operations on the upper extremity distal to the shoulder than on the shoulder (8.3 (± 4.3) kg vs. 6.37 (± 3.39) kg; *p* < 0.001), hip (8.24 (± 4.4) kg vs. 6.37 (± 3.39) kg; *p* = 0.003) or spine (9.11 (± 4.59) kg vs. 6.37 (± 3.39) kg; *p* = 0.005). There were significant differences in terms of the amount of recyclable material (x^2^(5) = 15.45; *p* = 0.009). Operations on the upper extremities continued to generate significantly less recyclable waste than operations in the shoulder area (0.77 (± 0.20) kg vs. 1.19 (± 0.36) kg; *p* = 0.037) or the hip (0.77 (± 0.20) kg vs. 1.3 (± 0.58) kg; *p* = 0.012).

### Type of operation

**Fig. 2:**
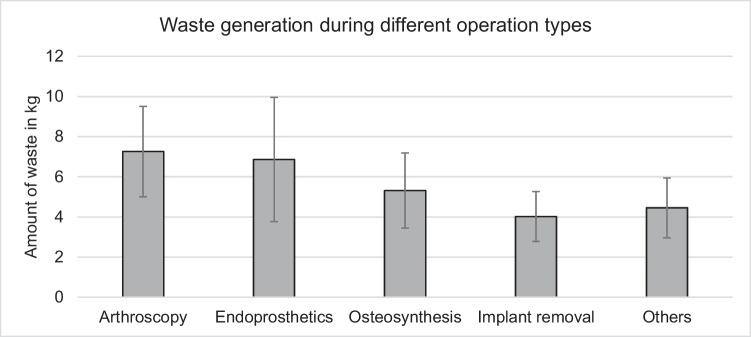
Waste generation during different operation types: The amount of waste differed significantly between different types of operations (*p* < 0.001). When comparing the amount of waste generated per minute of operation, no significant differences were found

Analysing all operations regarding the different types of operations, significant differences in total waste (x^2^(6) = 63.54; *p* < 0.001), separate hospital-specific waste (x^2^(6) = 43.58; *p* < 0.001) recyclables (x^2^(6) = 69.23; *p* < 0.001) and reusable drapes (x^2^(6) = 27.39: *p* < 0.001) where found Fig. 2. Implant removals produced significantly less total waste on average compared to all the other operation types (4.02 (± 1.59) kg vs. 5.59 (± 2.34);*p* = 0.002), both in terms of hospital-specific waste and recyclables, whereas arthroscopic procedures generated significantly more waste than open procedures(7.32 (± 2.25) kg vs. 5.15 (± 2.17) kg; *p* < 0.001). Endoprosthetic operations also produced significantly more waste than all the other types (6.99 (± 3.19) vs. 5.25 (± 2.10) kg; *p* < 0.001).

### Duration of operation

**Fig. 3:**
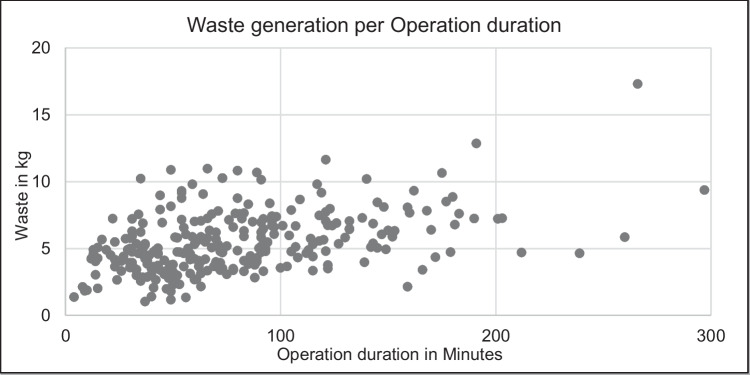
Waste generation per operation duration: The amount of waste significantly positively correlated with the duration of the operations. Longer operating times resulted in a significantly higher amount of total waste (*p* < 0.001)

The duration of all operations from incision to suture was significantly different between the individual types of operation (x^2^(6) = 61.90; *p* < 0.001) and the anatomical region (x^2^(5) = 23.80; *p* < 0.001). The amount of hospital-specific waste significantly positively correlated with the amount of recyclables (*p* < 0.001) and with the weight of reusable drapes (*p* = 0.001). In addition, longer operating times resulted in a significantly higher amount of total waste (*p* < 0.001) Fig. 3, hospital-specific waste (*p* < 0.001) and recyclables (*p* < 0.001) whereas the weight of reusable drapes was not depended on the duration of the operation. However, a larger amount of reusable drapes resulted in an increased proportion of recyclables in relation to the total amount of waste (*p* < 0.001) and a significantly longer duration between the start of procedures and the incision (*p* < 0.012).

When comparing the amount of waste generated per minute of operation, no significant differences were found between the types of operation (x^2^(6) = 3.5; *p* = 0.744) or anatomical regions (x^2^(5) = 2,99; *p* = 0,701). The average amount of waste per minute of operations was 94 (± 68) g.

## Discussion

This study identifies anatomical region, type and durations of operation as significant determinants of waste generation in operating rooms. The anatomical region of operation influenced the amount of drapes and thus the waste, when using disposable drapes. The type of operation seemed to indirectly influence the amount of waste through the duration of the operation.

The total amount of waste of approximately 5.45 kg per operation with 19.72% recyclable material was consistent with other studies that examined waste amounts for a general range of orthopaedic and trauma surgery operations [14–17]. Overall, large differences exist among previous studies, which could be attributed to the small number of operations evaluated. In contrast, this study is characterized by a high number of 272 operations and the mixed orthopaedic and trauma surgery spectrum of a maximum care provider and Level I trauma centre at a university hospital.

### Operation regions as a determinant influencing waste generation

This study showed that the anatomical region of operation appears to have a significant influence on waste. Since disposable drapes correspond to hospital-specific waste due to the risk of infectious contamination [7], differences in the amount of waste reflect variations in draping requirement. When reusable drapes were used, there were no longer any significant differences between different operation areas in terms of hospital-specific waste, but there were differences in terms of discarded reusable drapes, suggesting that the differences between operation areas are due to the amount and type of drapes used. The amount of recyclable material also varied between anatomical regions, which can be attributed to the sold recyclable packaging of reusable drapes. In this study, the use of reusable drapes made it possible to consider drapes and the remaining waste separately. A significantly higher amount of drapes were used in operations upon shoulder or hip than upon the rest of the upper or lower extremity, most likely due to the more complex draping techniques and higher number of drapes required, although the duration of the operation, which varies depending on the anatomical region, also appears to have an impact on the amount of waste. This might explain the results of other studies that described significant differences of the amount of waste between operations on the hip and on the hand or foot [14, 16]. This study also measured higher amounts of drapes in spinal operations, which can be attributed to standardized multi-layer draping to accommodate intraoperative imaging devices within the sterile field at this clinic. This may also explain the large variance in waste amounts across studies on the same types of operation. When considering only endoprosthetic operations, other studies have reported variable waste quantities ranging from 7 kg, as in this study, to over 16 kg per operation [13, 14, 17, 18]. Local draping standards may have a significant impact on waste generation when double drapes are used or replaced during the operation. This dependence of waste volumes on location conditions and operating room staff has been described previously [17, 19, 20]. Effective sterile coverage of the operation area, avoiding unnecessary double draping and increasing the use of reusable textiles, should therefore be evaluated especially for operational areas with high waste volumes.

### Operation types and duration as a determinant of waste generation

In addition to the drapes that need to be reprocessed, in this study, the amount of waste appears to depend significantly on the type and duration of the operation. The volume of waste differed significantly between different types of operation, regardless of the drapes used. Endoprosthetic and arthroscopic operations produced significantly more waste than osteosynthesis, implant removal or soft tissue procedures independent of the anatomical region. The duration of these procedures also varied significantly, and a significant correlation was observed between the amount of waste produced and the duration of the operation, both in terms of hospital-specific waste and recyclable materials. However, when calculating the amount of waste generated per minute of the operation, no significant differences remained between the types of procedure or anatomical region. These findings suggest that overall waste generation is largely driven by the duration of the operation. The observed results are consistent with other studies that have evaluated larger amounts of hospital-specific waste and recyclable materials in arthroscopic and endoprosthetic operations compared to others types of operation [14, 21]. This correlation explains the differences in waste volumes between primary and revision arthroplasty in the same operation area, as revision operations typically involve longer operation times [18, 22]. A correlation between waste volume and operating time has already been observed in general surgery [20]. This study demonstrates this correlation for the first time in orthopaedic and trauma surgery.

### Operating room management in the context of sustainability

In the context of a more sustainable healthcare system, waste management is a crucial factor alongside energy and water management. Particularly in operation areas, where high energy consumptions and significant waste volumes contribute to high greenhouse gas emissions, there is great potential for improving the ecological balance through targeted measures [11, 12, 23].

Hospital-specific waste is a key aspect in the development towards more sustainable operations due to the greenhouse gas-intensive disposal processes involved. The separation of recyclable materials and there reintroduction into the raw materials cycle has already been identified as an effective and easy-to-implement option for reducing hospital specific waste [18, 24, 25].

In addition to effective waste separation with recycling potential, the use of reusable materials represents a valuable opportunity for further waste reduction and, through reuse, also reduces emissions from the production of new materials, such as bed covers and caps [26, 27] and reusable instruments, that are resterilised for reuse are now standard practice in many hospitals [9, 28, 29]. Disposable textiles are classified as hospital-specific waste due to contamination with body fluids and tissue and are therefore also responsible for relevant greenhouse gas emissions due to their energy-intensive disposal [7]. In this regard, reusable gowns have already distinguished themselves in several studies through a convincing life cycle assessment [23, 30, 31] and high level of comfort for staff [32]. Reusable drapes have also proven themselves through a significant reduction in waste volumes and greenhouse gas emissions, as well as high acceptance by operating room staff [33].

In addition to recycling, reusable materials and reuse, further strategies including reducing double packaging, minimizing excessively individually packaged instruments and materials and optimizing well-packed operational kits [1, 11, 34]. Effective team communication is also essential to avoid materials being opened unnecessarily or incorrectly [35, 36].

Energy management for heating, air conditioning, light and ventilation [19, 23, 37] and the selection and duration of anaesthetic gas use [7, 38] are additional factors alongside waste management. The duration of the operations has a relevant influence not only on the amount of waste produced but also on the amount of energy consumption and anaesthetic gas emissions. Efficient coordination, clear communication and precise workflows are essential to minimize operation times, not only from an economic but also ecological perspective.

Beyond these factors, additional aspects play a decisive role in making operational disciplines more sustainable. Ultimately a holistic concept that takes ecological, economic and social aspects into account is the key to a sustainable healthcare system and simple measures often have the greatest overall impact [11, 39, 40]. Studies and detailed analyses such as this one are essential for establishing a scientific foundation. They enable the identification of challenges for the development of targeted, evidence-based interventions.

## Data Availability

No datasets were generated or analysed during the current study.
